# Methylated Bone Morphogenetic Protein 3 (BMP3) Gene: Evaluation of Tumor Suppressor Function and Biomarker Potential in Biliary Cancer

**DOI:** 10.4172/2155-9929.1000145

**Published:** 2013-08-03

**Authors:** John B Kisiel, Jia Li, Hongzhi Zou, Abdul M Oseini, Benjamin B Strauss, Kadra H. Gulaid, Catherine D Moser, Ileana Aderca, David A Ahlquist, Lewis R Roberts, Abdirashid M Shire

**Affiliations:** Division of Gastroenterology and Hepatology, Mayo Clinic, Rochester Minnesota, USA

**Keywords:** Cholangiocarcinoma, Gallbladder neoplasms, Early detection of cancer, Biological markers

## Abstract

**Background:**

Although cholangiocarcinoma (CC) is an uncommon and highly lethal malignancy, early detection enables the application of potentially curative therapies and improves survival. Consequently, tools to improve the early diagnosis of CC are urgently needed. During a screen for genes epigenetically suppressed by methylation in CC that might serve as methylation markers for CC, we found that the *BMP3* gene is methylated in CC cell lines, but the potential diagnostic value and the function of *BMP3* in CC are unknown.

**Methods:**

We aimed to quantitatively assess *BMP3* methylation in resected CC tumor specimens using methylation specific PCR and evaluate the tumor suppressor role of *BMP3* in biliary cancer cell lines in comparison to an immortalized normal cholangiocyte cell line. Expression of BMP3 was quantified by mRNA levels before and after treatment with 5-Aza-2’-deoxycytidine and trichostatin A. After transfection with a *BMP3*-containing plasmid, cell viability was measured using the bromodeoxyuridine incorporation assay and apoptosis quantified by caspase assay.

**Results:**

In primary CC tumor tissue specimens significantly more methylated *BMP3* copies were found when compared to matched benign bile duct epithelium from the same patient, with high specificity. *BMP3* expression was absent in cell lines with *BMP3* methylation; this suppression of *BMP3* expression was reversed by treatment with a DNA demethylating agent and histone de-acetylase inhibitor. Transfection of a *BMP3*-expressing construct into a *BMP3*-negative biliary cancer cell line restored *BMP3* mRNA expression and reduced cell proliferation and cell viability while increasing the rate of apoptosis.

**Conclusion:**

These findings strongly support a tumor suppressor role for *BMP3* in CC and suggest that *BMP3* methylation may be a new biomarker for early detection of CCs. of the peptidome are also involved.

## Introduction

Cholangiocarcinomas (CCs) are malignant tumors of cholangiocytes, the epithelial cells of the bile ducts. Worldwide, CC accounts for 3% of all gastrointestinal cancers [1]. In the US, the recently reported annual incidence of intra- and extra-hepatic cholangiocarcinoma and gallbladder cancer combined is up to 8 cases per 100,000 (up to 25,000 new cases annually), with a higher incidence above age 65 [2-4]. Approximately 60% of CCs are extrahepatic (ECC), arising from the region of the bifurcation of the right and left hepatic ducts extending distally to the distal common bile duct [4,5]. The remaining 40% of CCs are intrahepatic (ICC), arising in the bile ducts within the liver [4]. Risk factors for the development of CCs include congenital biliary cysts, primary and secondary sclerosing cholangitis (PSC), liver fluke infestation, hepatitis B and hepatitis C virus infections, metabolic syndrome, alcohol consumption, cirrhosis, diabetes and smoking [6-10]. Many patients lack an identifiable risk factor other than age, making early diagnosis of CC extremely difficult [11]. Consequently, the majority of patients with CC present at an advanced stage and the overall 5-year survival of patients with CC is only 8-12% [3,4]. However, survival is substantially better in patients diagnosed with early stage ICC and in those with ECC for whom surgical resection or combined neoadjuvant chemoradiation and liver transplantation are potentially curative [12-15].

There is therefore an urgent need for tools capable of making an early diagnosis of CC in pre-symptomatic individuals. Epigenetic suppression of tumor suppressor gene expression by gene methylation is a well-known mechanism of early carcinogenesis, tumor promotion, and metastasis which has been exploited as a biomarker of malignant transformation. Limited promoter methylation profiles of a number of tumor suppressor genes have been reported in intrahepatic and extrahepatic cholangiocarcinoma [16-18]. However, comprehensive studies for DNA methylation as a biomarker and mechanism for regulation of gene expression in CC have been lacking until recently.

Panels of novel target genes which are regulated by DNA hypermethylation and have potential utility as biomarkers have been identified. In a recent study of a methylated biomarker panel in CC tissues, the combined sensitivity was 87% at 100% specificity [19]. Of the genes in the panel, three novel genes (*CDO1, DCLK1*, and *ZSCAN18*) were found to be frequently methylated in CC in addition to *SFRP1*, which has been previously reported [20]. Additional methylation markers have also been identified in tissues (*CCND2, CDH13, GRIN2B, RUNX3*, and *TWIST1*) and tested in bile samples of ECC patients and controls, the 5-gene panel showed a sensitivity of 83% [21]. While new DNA methylation profiling tools such as methyl-sequencing and methyl-DNA immunoprecipitation arrays [22] are expected to identify many new markers, the mechanistic understanding of the role of methylation of these genes in biliary carcinogenesis remains poor. For a limited number of established candidate genes including *SFRP1* it has been demonstrated that DNA methylation within gene promoters inactivates transcription, [16] suggesting a tumor suppressor mechanism in CC.

*Bone Morphogenetic Protein 3 (BMP3)* has been shown to be methylated in cancers of the colorectum, pancreas, stomach, lung and breast, [23-28] but has not been reported in biliary cancers. Bone morphogenetic proteins constitute a large subgroup within the transforming growth factor beta (TGF-ß) superfamily. The approximately 30 members of the *BMP* subfamily were originally identified as inducers of bone formation, but *BMP*s are also now known to be involved in a variety of developmental processes [29]. There is an increasing recognition of the tumor suppressor function of *BMP3*. In the CpG island methylator phenotype of colon cancer, promoter methylation of *BMP3* is strongly associated with the *BRAF* V600E mutation [30]. In studies of colon cancer cell lines, *BMP3* was down-regulated by a methylation-dependent mechanism. Further, forced re-expression of *BMP3* led to suppression of colony formation, supporting a tumor suppressor function for *BMP3* [23].

We therefore hypothesized that *BMP3* expression may be reduced in biliary cancers, resulting in a lack of tumor suppressor function due to aberrant *BMP3* promoter methylation, and that methylated *BMP3* may serve as a clinically useful biomarker in these cancers. Accordingly, our goals were to: 1) perform a preliminary assessment of the diagnostic value of methylated *BMP3* by comparing methylated *BMP3* copy numbers in DNA samples taken from surgically resected human CC tissues in comparison to benign bile duct and colonic mucosal tissues; 2) demonstrate that methylation silences *BMP3* expression in biliary cancer cell lines, by examining *BMP3* mRNA levels after treatment of biliary cancer cell lines with a DNA methylation inhibitor alone or in combination with a histone deactylase inhibitor; and 3) demonstrate a functional tumor suppressor role for *BMP3* in biliary cancers by assessing the effect of transfecting a functional *BMP3* plasmid construct on cell proliferation and apoptosis of biliary cancer cell lines.

## Materials and Methods

### Cell lines and primary tissues

Five malignant biliary cancer cell lines were used in this study, KMC-1 (derived from an ICC), KMCH-1 (derived from a mixed hepatocellular carcinoma (HCC)/CC, but having a primarily biliary phenotype), KMBC and OZ (derived from ECCs), and Mz-ChA-1 (derived from a gallbladder adenocarcinoma) [31-35]. In addition, H-69, an SV40-transformed immortalized normal cholangiocyte cell line, was used as a control [36]. H-69 cells were grown in DMEM and DMEM/Ham F-12 supplemented with 10% FBS, adenine, insulin, epinephrine, T3-T, epidermal growth factor (EGF), hydrocortisone and antibiotics. The other cell lines were cultured in Dulbecco’s modified Eagle’s medium (Invitrogen), supplemented with 1% penicillin and 10% (v/v) fetal bovine serum. Cell lines were maintained in a humidified incubator at 37°C and 5% CO_2_.

Tissue samples of primary tumors were selected from patients who had undergone segmental hepatic resection or pancreaticoduodenectomy at Mayo Clinic (Rochester, MN) with an archived surgical specimen and a confirmed pathologic diagnosis. The study was approved by the Mayo Clinic Institutional Review Board. For each patient with CC, tissues from the primary CC tumor were compared with matched non-malignant bile duct epithelial control samples from their own resection specimen. Clinical variables abstracted included the type of CC (ICC vs. ECC), the presence of PSC, gender, age at the time of resection, and the carbohydrate antigen 19-9 level (CA 19-9, in units per milliliter, U/mL).

### Nucleic acid extractions

Genomic DNA and RNA were extracted from cell lines and primary tissues using the DNeasy Tissue Kit and RNeasy Kit (Qiagen, Valencia, CA). DNA was bisulfite treated using the EZ DNA Methylation Kit (Zymo Research, Orange, CA) and eluted in buffer.

### Methylation-Specific PCR (MSP)

**Figure 1:**
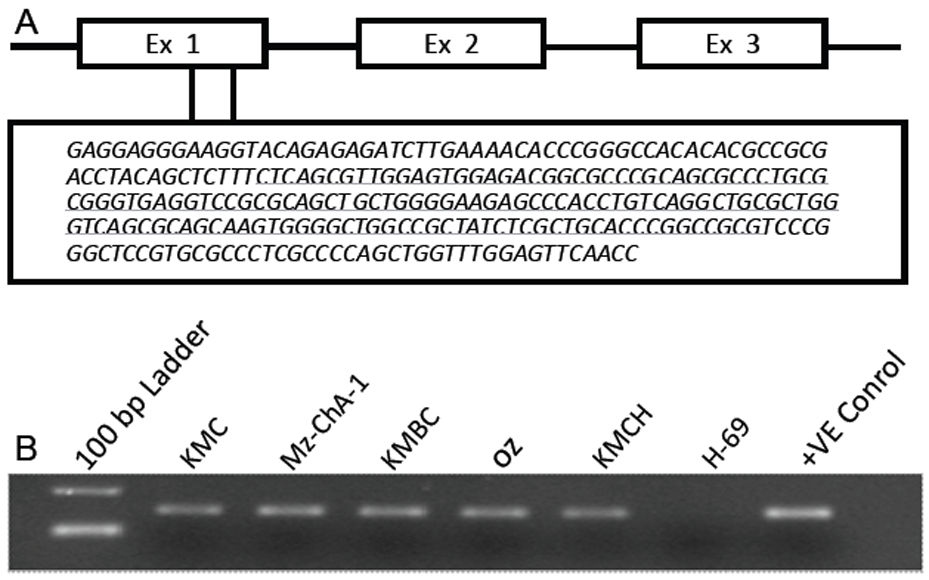
(A) CpG island targeted in exon 1 of the *BMP3* gene. (B) *BMP3* is methylated by MSP in all five biliary cancer cell lines but not in the immortalized normal cholangiocyte cell line H-69.

Methylation of CpGs in a region of the first exon of *BMP3* (Figure 1) was assessed by MSP in the five malignant biliary cancer cell lines KMC-1, KMCH-1, KMBC, OZ, and Mz-ChA-1, and the immortalized normal cholangiocyte cell line H-69. One μl of bisulfite-modified DNA was amplified in a reaction containing 1×PCR buffer (Applied Biosystem, Branchburg, NJ), 1.5 mM MgCl_2_, each dNTP (Roche Diagnostics, Indianapolis, IN), each of the methylation specific primers and AmpliTaq Gold polymerase (Applied Biosystem). The forward and reverse primer sequences were 5’-TTTAGCGTTGGAGTGGAGACGGCGTTC-3’ and 5’-CGCGACCGAATACAACGAAATAACGA-3’, respectively. CpGenome™ Universal Methylated bisulfite-treated human genomic DNA DNA (Chemicon, Billerica, MA) was used as a positive control for MSP.

### Bisulfite genomic sequencing

DNA from the KMC-1, Mz-ChA-1, and H-69 cell lines was bisulfite treated. One μl bisulfite-modified DNA was amplified in a reaction containing SYBR Green Supermix (Bio-Rad) and each primer. The forward and reverse primers were 5’-GAGGAGGGAAGGTATAGATAGA-3’ and 5’-AATTAAACTCCAAACCAACTAAAAC-3’, respectively. PCR products were cut from gels and purified using QIAquick Gel Extraction Kit (Qiagen), and then ligated into pCR 2.1-TOPO cloning vector using a TOPO TA Cloning Kit (Invitrogen, Carlsbad, CA). Ten colonies from each cloning reaction were grown in LB medium. The plasmids were extracted using QIAprep Spin Miniprep Kit (Qiagen) and then sequenced on an ABI Prism 377 DNA Sequencer (Perkin Elmer, Boston, MA) to obtain the detailed methylation status of each CpG site.

### Real-time methylation-specific PCR (RT-MSP)

The DNA concentration of each sample was quantified using Quant-iT PicoGreen (Invitrogen; Carlsbad, CA). The DNA was then bisulfite-treated using the EZ DNA Methylation Kit (Zymo Research, Irvine CA). One μl bisulfite-treated DNA was used as a template for methylated DNA quantification with a fluorescence-based real-time polymerase chain reaction (PCR). The forward and reverse primers were 5’ TAA TTT TCG GTT TCG TCG TC 3’ and 5’ AAA AAA ACA ACCC TAC TCG CC 3’ respectively (IDT, Coralville, IA). A region without cytosine-phosphate-guanine sites in the *β-actin* gene was also quantified with real-time PCR using primers recognizing the bisulfite-converted sequence as a reference for bisulfite treatment and input DNA. Forward and reverse primers were 5’ TTT TTT TTG GTG TTT GTT TTT TTG ATT A 3’ and 5’ CAC CAA CCT CAT AAC CTT ATC ACA C 3’ respectively. PCR reactions for tissue DNA samples were performed with 12.5 μl of SYBR Green MasterMix (Roche, Mannheim, Germany). All reactions were run on Roche 480 LightCyclers (Indianapolis, IN). For all plates bisulfite-treated CpGenome Universal Methylated DNA (Millipore) was used as a positive control and serially diluted to create standard curves for calculation of copy number of methylated *BMP3* and *β-actin*. Copy numbers of each PCR product were corrected by the concentration of DNA in the input sample, prior to bisulfite treatment. Unmethylated copies were not targeted for amplification.

### Treatment with 5-Aza-2’-deoxycytidine and trichostatin A

The KMC-1 and Mz-ChA-1 cell lines were split to low density in 5 ml flasks and were grown in Dulbecco’s modified Eagle’s medium supplemented with 10% fetal bovine serum for 24 hrs. The cell lines were then either treated with 5 μM of the DNA methylation inhibitor 5-Aza-2’-deoxycytidine (5-Aza-dC) or mock treated with DMSO for 5 days. The medium containing 5-Aza-dC or DMSO was changed every 24 h. The dose and timing of 5-Aza-dC was based on prior optimization tests [37]. In addition, cells treated with 5-Aza-dC were further treated with 0.5 μM of the histone deacetylase inhibitor trichostatin A for an additional 24 hours.

### Quantitative real-time reverse transcription-PCR

*BMP3* mRNA expression of the CC cell lines with or without 5-Aza-dC treatment and of vector or BMP3-transfected cell-lines was quantified by real-time RT-PCR. Reverse transcription was performed on 1 μg of total RNA using the Omniscript RT Kit (Qiagen). One μl cDNA was amplified using the Applied Biosystems 7300 Real-Time PCR System (Foster City, CA). The samples were amplified in a reaction containing SYBR Green Supermix (Bio-Rad) and each primer (HS 0060963_m1) (Applied Biosystems, Foster City, CA) using 18 S RNA as an internal reference.

### Functional assays for cell proliferation, cell viability and cell death

The Mz-CHA-1 cell line was used for functional assays of the effect of BMP3 expression on biliary cancer cells. The sequence of the *BMP3* cDNA clone (Origene, Rockville, MD) was confirmed by restriction digestion and DNA sequencing. A second construct expressing the full-length *BMP3* cDNA was prepared in our laboratory by cloning into the expression plasmid pcDNA3.1. The expression plasmid also contained a FLAG-tag and was verified by sequencing. Cells transfected with control vector or with a plasmid expressing BMP3 were assessed to determine the functional role of this gene.

The day before transfection, Mz-ChA-1 cells were plated at a density of 4×10^5^ cells/well in six-well plates and 1.5×10^4^ cells/well in 96-well plates. They were then transiently transfected overnight using a mixture of either pcDNA 3.1 *BMP3* or pcDNA3.1 vector plasmid and Fugene 6 transfection reagent (Roche, Indianapolis, IN) according to the manufacturer’s instructions. The final ratio of Fugene 6 to DNA was 3:1. Two days after transfection, cells were examined for the expression of green fluorescent protein (GFP) by fluorescence microscopy (Nikon Eclipse TS100, Tokyo, Japan) to confirm that the cells were successfully transfected.

Following transfection, Mz-ChA-1 cells were assayed for viability and proliferation. To investigate the effect of forced expression of *BMP3* on MzChA-1 cell growth, we quantitated cell viability by the MTT [3-(4,5-dimethylthiazol-2-yl)-2,5-diphenyl-tetrazolium bromide] colorimetric assay and cell proliferation by the bromodeoxyuridine (BrdU) incorporation assay. For the MTT assay, Mz-ChA-1 cells were seeded into 96-well plates 48 hours after transfection, each well was then supplemented with 10 μl MTT (Sigma Aldrich) and incubated for 4 h at 37°C. The medium was then removed, and 150 μl DMSO (Sigma Aldrich) was added to solubilize the MTT formazan. The absorbance was measured at 570 nm using an enzyme-linked immunosorbent assay (ELISA) plate reader (μQuant, Bio-Tek Instruments, USA). The viability of vector-transfected control cells was set to 100%, and the viability of *BMP3*-transfected cells was expressed as a percentage of formazan absorbance compared with that of control cells. The BrdU incorporation assay was performed according to the manufacturer’s instructions (Roche, Indianapolis, IN). Each experiment was performed in ten replicates at least three times.

The effect of *BMP3* expression on the apoptosis of CC cells was assessed by caspase-3/7 activity. Mz-ChA-1 cells were seeded into 96-well plates. After 48 hours, the homogeneous caspase-3/7 reagent (Promega, Madison, WI, U.S.A.) was added in a 1:1 ratio of caspase reagent to cell culture medium. After shaking on a plate shaker for 3 hours at room temperature, fluorescence was measured at an excitation wavelength of 485 nm and emission wavelength of 520 nm on an FLx800 Microplate Fluorescence Reader (BIO-TEK Instruments Inc., Winooski, VT).

### Statistical considerations

#### Cell line studies

All data are expressed as means and standard errors of the mean. Differences between groups were compared using an unpaired, two-tailed t test.

#### Tissue study

The methylation level for each candidate gene was defined as the absolute copy number of methylated target sequences after PCR amplification, expressed as a continuous variable. Based on the mean difference and standard deviation of methylated *BMP3* copy numbers in a recent comparison of pancreatic cancer samples to normal colon samples, [25] we estimated that 12 samples in each group would provide greater than 80% power to detect a difference in mean methylation greater than 400 copies in each group in a one-sided test at the 5% significance level. Given the small sample size, a non-parametric comparison for paired data, the Wilcoxon signed-rank test, was used. Logistic regression was used to model the strength of association between methylated *BMP3* copy number and CC and to construct a receiver operating characteristics curve (ROC). From the ROC, point estimates for sensitivity (with 95% confidence intervals, CI) were calculated by the efficient score method after setting specificity to 100%. Analyses were performed in JMP version 9.0.1 (SAS Institute, Cary, NC).

## Results

### *BMP3* is specifically methylated in biliary cancer cell lines

**Figure 2:**
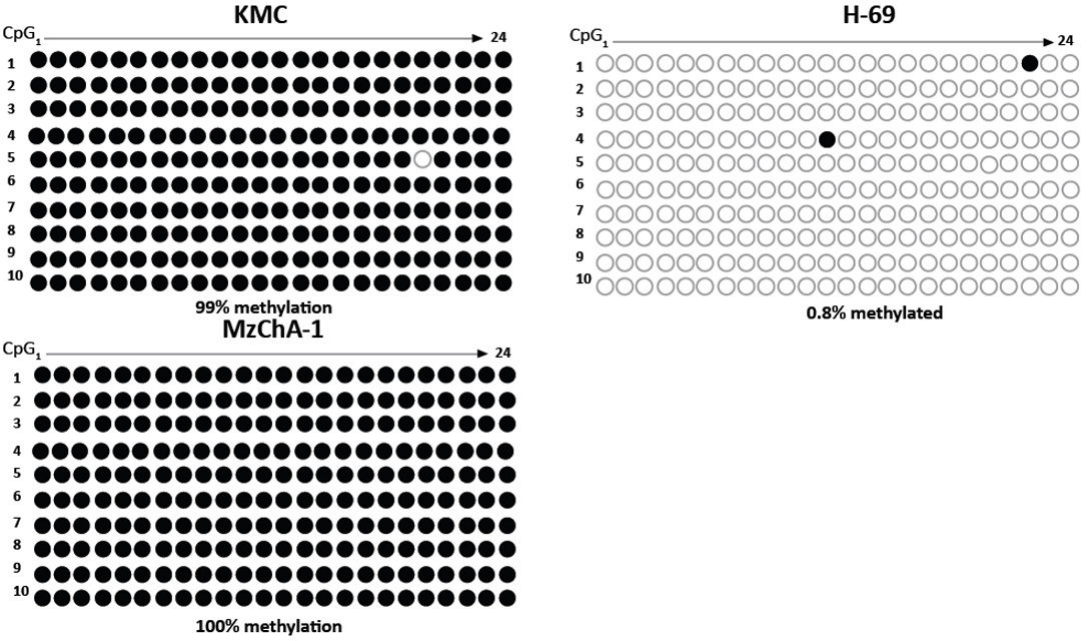
Bisulfite genomic sequencing methylation pattern of CpG sites for the targeted *BMP3* island in ten clones from each of two malignant biliary cancer cell lines and the normal cholangiocyte cell line.

As shown by amplification in MSP reactions, CpG sites in the targeted island (Figure 1A) were methylated in genomic DNA from all five biliary cancer cell lines, but not in DNA from the H-69 immortalized normal cholangiocyte cell line (Figure 1B). Further, to map the exact methylation pattern of the CpG sites in the targeted island, three cell lines, KMC-1, Mz-ChA-1, and the H-69 control cell line were selected for bisulfite genomic sequencing (BGS). The two biliary cancer cell lines showed 100% CpG methylation within the CpG island for all 10 clones tested per cell line, while there was less than 1% methylation in the CpG island of the normal cholangiocyte cell line (Figure 2).

### BMP3 is specifically methylated in CC tumor tissues

Real-time methylation specific PCR (RT-MSP) quantified the number of methylated *BMP3* promoter copies in 12 resected primary human CCs in comparison to matched benign bile duct tissues. The median age of the patients at the time of the resection was 61 years, with an interquartile range (IQR) of 51-75 years (Table 1). The majority (10/12, 83%) were women and most (10/12) had intrahepatic tumors. None of the patients had PSC or cirrhosis.

**Figure 3:**
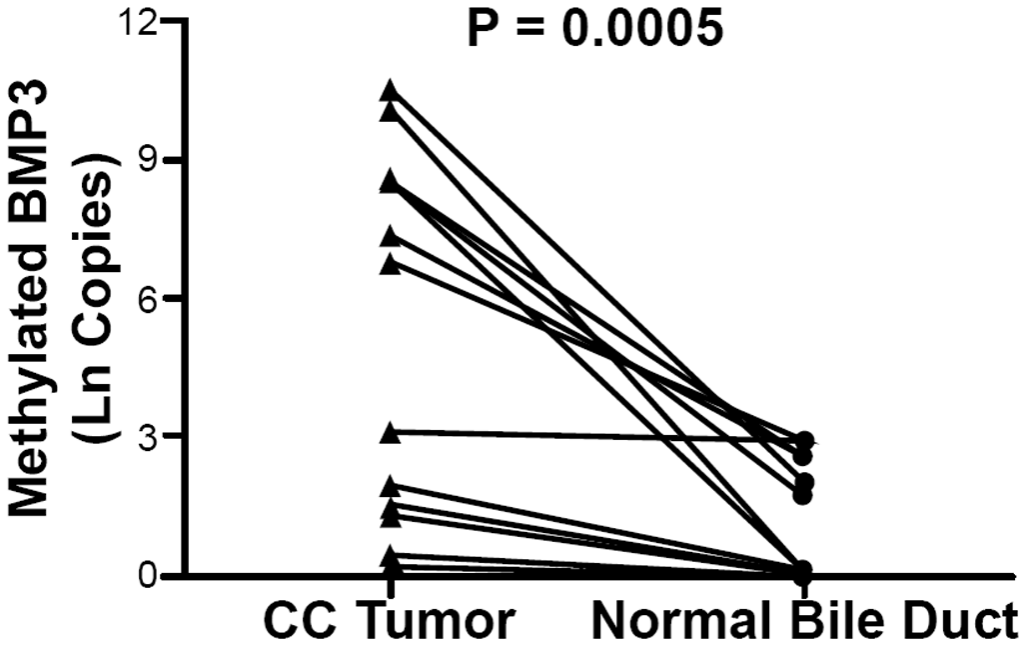
Natural logarithm transformed copy numbers of methylated *BMP3* target sequences in cholangiocarcinoma (CC) tissue and paired benign bile duct epithelium samples from 12 patients

*β-actin* amplified in all samples. The median number of methylated *BMP3* copies in the 12 CC tissues was 446 (range, 0-37,000); in contrast, the median in the matched non-malignant bile duct samples was 0 copies (range, 0-17) (p < 0.003). Natural logarithm transformed copy numbers are displayed in Figure 3. Results were not significantly different when corrected by *β-actin.* At 100% specificity, methylated *BMP3* was 58% sensitive (95% CI, 29 - 84%) for CC in comparison to non-malignant bile duct tissues. The AUC was 0.83, indicating strong association.

### Treatment with DNA methylation and histone deacetylase inhibitors restores *BMP3* mRNA Expression

**Figure 4:**
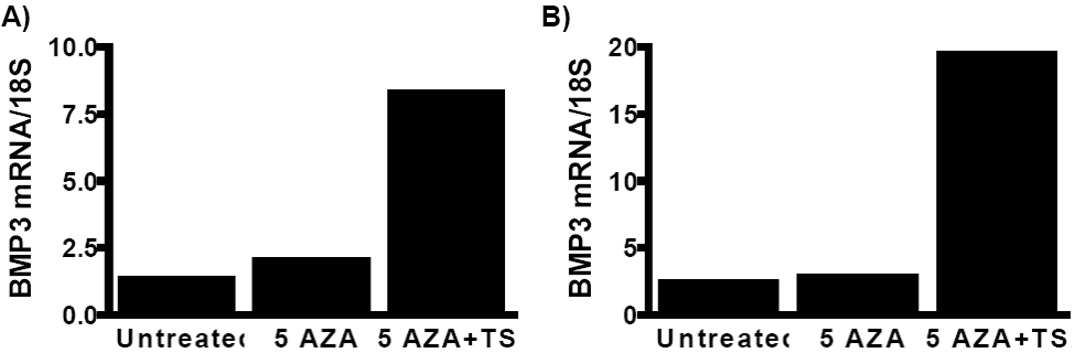
Treatment with the DNA demethylating agent 5-Aza-2’-deoxycytidine (5-AZA) and the histone de-acetylase inhibitor trichostatin A (TSA) derepresses *BMP3* gene expression in (A) KMC-1 cells and (B) Mz-ChA-1 cells.

The mRNA expression of BMP3 in the two biliary cancer cell lines KMC-1 and Mz-ChA-1 was largely suppressed at baseline. Following 5 days of 5-Aza-dC treatment alone, the expression of the *BMP3* mRNA was not significantly restored in either cell line, as measured using quantitative real-time reverse transcription-PCR. However, after an additional day of incubation with TSA the expression of the *BMP3* mRNA was increased 5-fold in the KMC-1 cell line and 8-fold in the Mz-ChA-1 cell line (Figure 4A & 4B).

### Forced expression of *BMP3* decreases cell proliferation and viability while increasing apoptosis of biliary cancer cells

**Figure 5:**
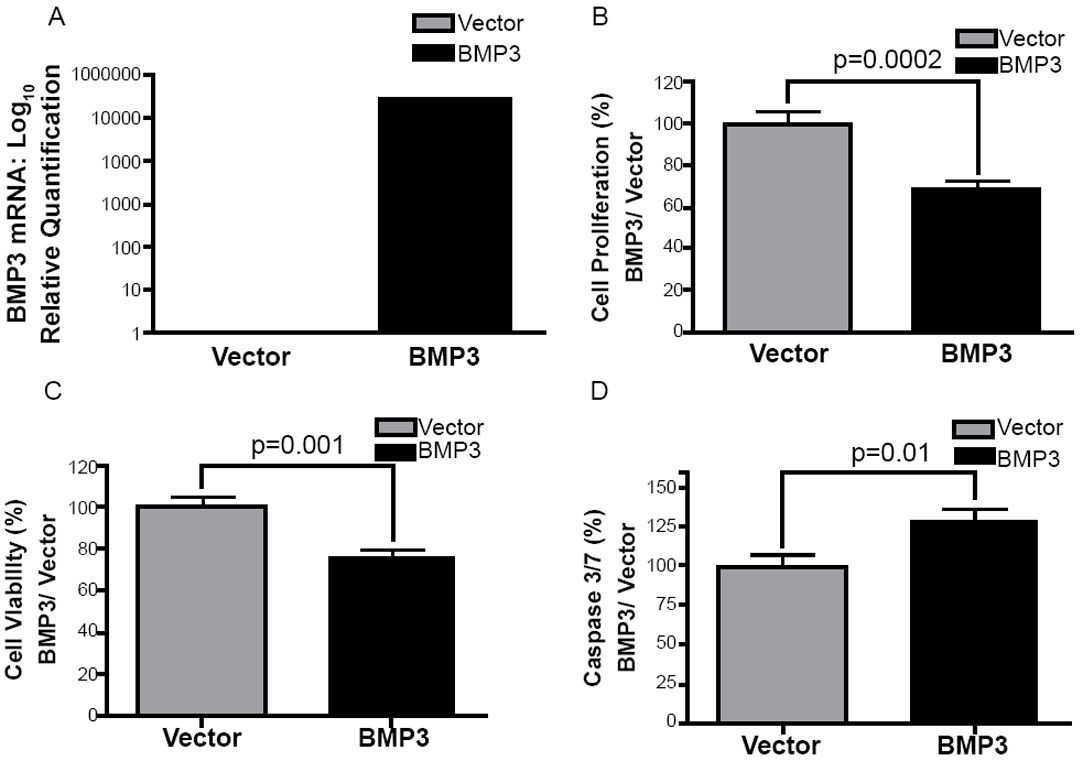
(A) *BMP3* gene expression in Mz-ChA-1 cells 48 hours after transfection with *BMP3* plasmid compared to control vector. (B) Forced expression of *BMP3* inhibits Mz-ChA-1 cell proliferation as assessed by the BrdU assay and (C) reduces Mz-ChA-1 cell viability as measured by the MTT assay. (D) Transfection with *BMP3* plasmid increased Mz-ChA-1 cell apoptosis at 48 hours, as measured by the relative fluorescence units in a caspase 3/7 assay.

Given the evidence for a tumor suppressor role for *BMP3* in other cancer types, we hypothesized that inactivation of *BMP3* expression in biliary cancer cell lines would be associated with increased cell proliferation and viability. Re-expression of *BMP3* in a biliary cancer cell line should therefore decrease cell proliferation and enhance apoptosis. Quantitation of *BMP3* expression by real-time PCR 48 hours after transfection showed a five-log increase in *BMP3* expression in Mz-ChA-1 cells transfected with a *BMP3* expressing plasmid construct, in comparison to vector transfected cells (Figure 5A). Forced expression of *BMP3* caused a 30% decrease in cell proliferation as measured by BrdU incorporation compared to controls (P=0.0002) (Figure 5B). Compared to vector control cells, expression of *BMP3* also reduced Mz-ChA-1 cell viability by 25%, as measured by the MTT assay (P=0.001) (Figure 5C). Forced expression of *BMP3* also significantly increased apoptosis in Mz-ChA-1 cells by 25% compared to vector controls, as measured by relative fluorescence units of caspase 3/7 (P=0.01; Figure 5D). *BMP3* expression therefore exerts a profound tumor suppressive effect on the Mz-ChA-1 biliary cancer cell line.

## Discussion

Our data strongly support a tumor suppressor role for *BMP3* in biliary cancers. The promoter region of *BMP3* was found to be methylated in all five biliary cancer cell lines tested, but not in an immortalized benign cholangiocyte cell line, and methylation of *BMP3* was associated with either low or absent *BMP3* gene expression in these biliary cancer cell lines. Combined treatment with the DNA methylation inhibitor 5-Aza-dC and the histone deacetylase inhibitor TSA restored *BMP3* mRNA expression. Moreover, forced re-expression of *BMP3* by transfection of a plasmid construct expressing *BMP3* in a biliary cancer cell line reduced cell proliferation and viability and increased apoptosis. This is the first report of a tumor suppressor function of *BMP3* in biliary cancer cell lines.

Results from cell lines were corroborated in tumor tissues. In resected CC tumor samples, we found a significantly greater number of methylated *BMP3* copies when compared to matched, non-malignant bile duct samples. This finding suggests that methylated *BMP3* may serve as a potential biomarker for cholangiocarcinoma, a disease for which there is a critical need for improved early detection of cancer.

*BMP3* is a powerful stool biomarker for the screening and diagnosis of colon cancer, [24,38] a disease in which *BMP3* has an established tumor suppressor role. In colorectal neoplasms with methylation in the *MLH1* promoter, bisulfite sequencing has demonstrated extensive hemi- or biallelic methylation of the *BMP3* promoter; in these tissues silenced expression of *BMP3* could be reversed by treatment with 5-Aza-dC and TSA [39]. Similar to our findings in biliary cancer cells, forced expression of *BMP3* in colorectal cancer cell lines also suppressed cell proliferation [23].

*BMP3* is also methylated in early stage colorectal cancers, and most importantly, in pre-cancerous tissue [23,38]. We have also reported frequent *BMP3* methylation in tissue and stool samples from patients with pancreatic cancers, even at early stage [25].

While CC tumor samples contain a significantly greater number of copies of methylated *BMP3* than benign bile duct tissues, the sensitivity was not high enough to support the use of *BMP3* as a single diagnostic marker for CC. The sensitivity was comparable to that found in other tissue sample studies in which single markers were 14-73% sensitive [16,20] and multi-marker panels were 74-91% sensitive [19-21]. In a multi-marker panel used for screening for colorectal cancer in stools, the high specificity of *BMP3* complements other more sensitive methylated and mutant genes [38].

The study has several limitations. The sample size of the tissue study was small but carefully calculated to assess the performance of a single marker in tissues while maximizing the use of scarce specimens. Tissues were not microdissected, and DNA from fibroblastic tumor desmoplasia and other stromal elements from normal controls were likely assayed as well. However, amplification of stromal DNA would be expected to dilute the methylated copy number, which remained substantial and significant in comparison to control tissues. While the study did not compare the accuracy of multiple markers in CC detection, the findings establish a tumorigenic mechanism for, and substantiate the clinical relevance of, *BMP3* methylation in CC. Treatment of cell lines with TSA after 5-Aza-dC increased *BMP3* expression to a greater extent than treatment with5-Aza-dC alone, suggesting that expression of BMP3 is dependent on histone acetylation as well as promoter methylation. The extent to which this could be tested was limited by the profound apoptotic effects of TSA on cell lines, preventing treatment with TSA prior to 5-Aza-dC [40].

In summary, this study provides compelling evidence for the importance of *BMP3* methylation in CC tumorigenesis. The frequent observation of methylated *BMP3* in CC tumor samples but not in matched benign bile duct epithelium suggests that DNA methylation markers are important targets for the development of a critically needed surveillance tool for patients at high risk for CC or a screening test for CC in the general population. Further studies are now indicated in biospecimens of patients at high risk for CC, such as those with PSC, to assess the value of *BMP3* and other methylation markers for screening or early detection of CC in biospecimens such as bile, bile duct brushings, blood or stool.

## Figures and Tables

**Table 1:** Clinical features of cholangiocarcinoma patients.

Characteristic	Result (n = 12)
Age at resection, Median (IQR), years	61 (51–75)
Female (%)	10 (83)
ICC (%)	10 (83)
PSC (%)	0
Cirrhosis (%)	0
Serum CA 19-9, median (IQR) U/mL	45 (5–214)

IQR, Interquartile range ICC, Intra-hepatic cholangiocarcinoma PSC, Primary sclerosing cholangitis U/mL, units per milliliter
